# Clinical features, predictive factors and outcome of hyperglycaemic emergencies in a developing country

**DOI:** 10.1186/1472-6823-9-9

**Published:** 2009-03-10

**Authors:** Anthonia O Ogbera, Jacob Awobusuyi, Chioma Unachukwu, Olufemi Fasanmade

**Affiliations:** 1 Department of Medicine, Lagos State University Teaching Hospital, Ikeja, Lagos, Nigeria; 2 Department of Medicine, University of Port Harcourt Teaching Hospital, Port Harcourt, Nigeria; 3 Department of Medicine, Lagos University Teaching Hospital, Idi-araba, Lagos, Nigeria

## Abstract

**Background:**

Hyperglycaemic emergencies are common acute complications of diabetes mellitus (DM) but unfortunately, there is a dearth of published data on this entity from Nigeria. This study attempts to describe the clinical and laboratory scenario associated with this complication of DM.

**Methods:**

This study was carried out in DM patients who presented to an urban hospital in Nigeria with hyperglycaemic emergencies (HEs). The information extracted included biodata, laboratory data and hospitalization outcome. Outcome measures included mortality rates, case fatality rates and predictive factors for HEs mortality. Statistical tests used are *χ*^2^, Student's t test and logistic regression.

**Results:**

A total of 111 subjects with HEs were recruited for the study. Diabetes ketoacidosis (DKA) and hyperosomolar hyperglycaemic state (HHS) accounted for 94 (85%) and 17 (15%) respectively of the HEs. The mean age (SD) of the subjects was 53.9 (14.4) years and their ages ranged from 22 to 86 years. DKA occurred in all subjects with type 1 DM and 73 (81%) of subjects with type 2 DM. The presence of HSS was noted in 17 (19%) of the subjects with type 2 DM.

Hypokalaemia (HK) was documented in 41 (37%) of the study subjects. Elevated urea levels and hyponatraemia were noted more in subjects with DKA than in those subjects with HHS (57.5%,19% vs 53%,18%). The mortality rate for HEs in this report is 20% and the case fatality rates for DKA and HHS are 18% and 35% respectively.

The predictive factors for HEs mortality include, sepsis, foot ulceration, previously undetected DM, hypokalaemia and being elderly.

**Conclusion:**

HHS carry a higher case fatality rate than DKA and the predictive factors for hyperglycaemic emergencies' mortality in the Nigerian with DM include foot ulcers, hypokalaemia and being elderly.

## Background

Diabetes mellitus is a chronic metabolic disorder that is gradually assuming pandemic proportions. It has been projected that this emerging scenario will be more evident in the developing world specifically African and Asian continents [1,2].

DM is often associated with a high disease burden especially in areas where there is poor accessibility to health facilities. Diabetes mellitus is one of the non communicable diseases associated with high case fatality rates in Africa[3].

Diabetic ketoacidosis (DKA) and hyperosmolar, hyperglycaemic state (HHS) are acute complications of diabetes mellitus that may be life threatening if not properly treated[4]. Diabetic ketoacidosis (DKA) – a triad of hyperglycaemia, hyperketonaemia and metabolic acidosis is the commonest hyperglycaemic emergency of DM [5-7] and often results in high morbidity and mortality rates. The treatment of HEs often involves the usage of significant healthcare resources[5].

In Nigeria, the brunt of the costs of health care provision is borne largely by the patients and family members. Given this scenario, the impact of an increasing prevalence of DM and its' associated complications in Nigeria will expectedly result in an unacceptably high disease burden.

HEs are important causes of DM deaths in Nigeria[3] hence we undertook to document the clinical, laboratory correlates and mortality rates associated with this all-important DM complication in our practice.

## Methods

This was a prospective study carried out in an urban hospital in Lagos, Nigeria with the study period spanning over six months. Patients with DM and those that had not previously been diagnosed with DM, aged above 12 years of age and presenting in hyperglycaemic emergencies were recruited for the study. The information extracted included biodata, plasma glucose and electrolye levels at presentation, duration of DM, previous treatment type prior to admission, and mortality data. The presence of ketones was determined by the detection of ketonuria using ketostix.

### Operational definitions

**Duration of DM **was categorized into short term duration, which referred to those that have had DM for less than 10 years, medium term duration, which referred to DM duration of 10–19 years and long term duration referring to DM duration of = 20 years

**Diabetes ketoacidosis **referred to blood glucose levels >13.8 mmol/L and the presence of metabolic acidosis (bicarbonate levels of <10 mmol/L-18 mmol/L) and or the presence of ketonaemia or ketonuria [8,9].

**Hyperosmolar hyperglycaemic state **referred to plasma glucose levels of >33.3 mmol/L and bicarbonate levels of >18 mmolL with or without the presence of ketonuria [8,9].

**Type 1 DM **– Type I DM referred to DM patients who have been on insulin since diagnosis and require insulin for survival.

**Type 2 DM **– Type 2 DM refers to patients with DM who were previously managed on sole lifestyle modification, or on oral hypoglycaemic agents. It also encompasses insulin requiring patients who initially were not insulin dependent.

Hypernatraemia and hyponatraemia referred to serum sodium levels of >145 mmol/L and < 136 mmol/L respectively[8].

Hypokalaemia and hyperkalaemia referred to serum potassium levels of <3.5 mol/L and >5 mmol/L respectively[8].

Urea levels were considered elevated if the blood urea nitrogen was >7.9 mmol/L[8].

The primary outcome measure was death and the predictive factors for this were studied. Mortality and case fatality rates for the hyperglycaemic emergencies were computed and compared in three age groups viz- the lowest recorded age up to 34 years (Young), those that were aged between 35 – 64 years (Middle aged) and those who were aged = 65 years (Elderly).

The Statistic tests used included Student's t test and *χ*^2 ^for analysis of quantitative and qualitative data respectively. The determinants of hyperglycaemic emergecncies' associated mortality were determined using logistic regression. Significance level was set at a p value <0.05. The Statistical package used for analysis was SPSS version 15.

Consent for the study was given by the Research and Ethics Committee of the hospital.

## Results

A total of 111 subjects with hyperglycaemic emergencies were studied. The subjects comprised 21 (19%)and 90 (81%) people with types 1 and 2 DM respectively.

DKA accounted for 94 (85%) and HHS for 17 (15%) of the hyperglycaemic emergencies.

### Biodata and clinical characteristics of subjects presenting with hyperglycaemic emergencies

The mean age (SD) of the study subjects was 53.9 (14.4) and their ages ranged from 22–86 years. The mean (age) of the females and that for males were comparable (51.8 (14.4) years vs 55.9 (14.3) years, p > 0.05). The mean (SD) age of subjects with type 1 DM was significantly lower than that of those with type 2 DM (40.5(17) years vs 57.1(11,7)years, p value > 0.001). The male: female ratio of subjects with type 1 DM was 1:1.3 while that of type 2 DM was 1: 1.2.

The mean blood glucose and duration of DM of the study subjects were 25.1 (8) mmol/L and 6.8 (9.4) years respectively. The mean (SD) plasma glucose for females and that for males were comparable (26.2 (8.6)mol/L vs 24.2 (7.3) mmol/L p > 0.05). The mean (SD) plasma glucose in subjects with types 1 and 2 DM were 28.2 (7.8)mmol/L and 24.5 (7.2)mmol/L respectively.

Hypertension was noted in 6 (29%) of subjects with type 1 DM and 28 (31%) of those with type 2DM but this difference was not statistically significant p > 0.05).

15 (14%) of the subjects did not have a prior history of DM and had the diagnosis of DM made at presentation with hyperglycaemic emergency.

DKA occurred in all subjects with type 1 DM (21) and 73 (81%) of subjects with type 2 DM. 17 (19%) subjects with type 2 DM had HHS.

Hypokalaemia was documented in 41 (37%) of the subjects with HEs and hyperkalaemia only in subjects with DKA -2 (2.1%). 11 subjects had hypotension 10%.

Other clinical features of the subjects with HEs are shown in Table 1

**Table 1:** Clinical characteristics of the subjects with hyperglycaemic emergencies

	DKA	HHS	P VALUE
Age	53.9(14.1)	54.1 (16.3)	>0.05
Sex (F:M)	41:53	11:6	0.001
Mean blood glucose	22.8 (5.9)	38.3 (3.7)	0.00001
Presence of HTN	29 (31%)	5 (30%)	>0.05
Altered mentation	18(19%)	8 (47%)	
Seizures	0	3(17%)	
DM duration	7 (9.8)	5.4 (7.3)	>0.05

The biochemical parameters of the subjects with DKA and HHS are compared in Table 2.

**Table 2:** Biochemical features of Hyperglycaemic emergencies

	DKA	HHS
Elevated urea levels	54 (57.4%)	9 (53%)
Hypernatraemia	2 (2%)	2 (12%)
Hypokalaemia	33 (35%)	8 (47%)
Hyponatraemia	18 (19%)	3 (18%)

The biochemical parameters of the subjects with DKA and HHS are compared in this table.

The lower the duration of DM, the higher the recorded number of HEs admissions and deaths. A large majority -89 (80%)- of the study subjects had short term duration of DM and the highest number of deaths-18 deaths-were recorded in this category of patients. The lowest number of admissions and deaths (7 and 1 respectively) were noted in subjects with long term duration of DM.

The morbidity pattern of hyperglycaemic emergencies is such that the greatest number of DKA and HHS related hospitalizations were recorded in the study subjects who were middle aged (62 (66%) vs 9 (53%).

Previously undiagnosed DM and poor drug compliance are responsible for over 50% of the precipitants of the hyperglycaemic emergencies. The precipitants of HEs are shown in Table 3.

**Table 3:** Precipitants of hyperglycaemic emergencies

Precipitating factor	No(Frequency)
Poor drug compliance	49 (44.1%)
Undiagnosed DM	15 (13.5%)
*DMFS	20 (18%)
**CVA	9(8.1%)
Sepsis	15 (13.5%)
≠ TDHS	3 (2.8%)

*diabetes mellitus foot syndrome,

**cerebrovascular accident,

≠ tropical diabetes hand syndrome

The mortality data showed that 23 of the subjects hospitalized with HEs died thus giving a mortality rate of 20%. Case fatality rates for DKA and HHS were 18% and 35% respectively. The male: female ratio of the HEs associated mortalities was 0.9:1.

The proportions of subjects with type 1 and type 2 DM that died were 6 (29%) and 17 (18%) respectively.

The major prognostic factors for hyperglycaemic emergencies' associated deaths include diabetes foot ulceration (DMFS) and low duration of DM. Other predictive factors for HEs associated mortality are shown in Table 4.

**Table 4:** Predictive factors for hyperglycaemic emergencies fatality

Variable	Odds ratio	95% CI
DMFS	2.9	0.86–9.68
Elderly	1.3	0.3–4.1
Hypokalaemia	0.5	0.2–1.6
Hypertension	0.3	0.1–0.9
Sepsis	3.2	0.6–16.7
Low duration of DM	1.7	0.1–16.0

## Discussion

Hyperglycaemic emergencies are oft encountered DM complications in clinical practice. They are the most serious acute metabolic complications of diabetes mellitus and are associated with excess mortality. The incidence of DKA in the United States of America is 4.6 and 8.0 per 1000 person-years among patients with diabetes, whereas that of HHS is less than 1 per 1000 person-years[10]. In sub-Saharan Africa data on incidence rates for the HEs are scarce. An earlier report by Ogbera et al[3] showed that hyperglycaemic emergencies account for 40% of all DM related hospitalization with a preponderance of DKA admissions compared to that of HHS.

Hyperglycaemic emergencies occur in all age groups of people with DM and in this report we found that DKA and HHS admissions were recorded more in the 36–64 years age bracket while the least number of DKA and HHS hospitalizations were found in those below 35 years of age. This study documented the mean age of subjects with HEs to be 54 years and this is lower than those reported from some Western, Asian sub-Saharan African countries [11-13]. In this report, the mean ages of subjects presenting with DKA and HHS [10,11] were comparable and these findings are diametrically opposed to some other reports[11,14] that showed a significant difference in the ages of people presenting with HHS and DKA. The noted difference between these reports and ours may be due to our inability to assess the C-peptide levels which would have helped in objectively classifying patients properly according to DM type. Of note in this report, is that gender was comparable in subjects presenting with HHS and those presenting in DKA.

Though precipitating factors for HEs are varied, infections, poor drug compliance and undiagnosed or new onset diabetes are factors that have consistently been reported as being associated with HEs [5,11]. Although our findings are consistent with the aforestated scenario, it is pertinent to note that poor drug compliance was a major precipitant of HEs in our study subjects. Documented reasons for poor drug compliance in our patients ranged from poor accessibility to health facilities, high costs of drugs often resulting from polypharmacy because of co-morbidities and also ignorance on self care habits of DM. Reported rates of undiagnosed diabetes presenting as HEs range from 10.9%–50%[7,11,12,15]. In this report undiagnosed DM accounted for 15% of the hyperglycaemic emergencies.

The low prevalence of hypertension of DM in our patients with type 2 DM may be explained by the fact the that some of the subjects were not previously known to have DM or hypertension and the presence of resultant hypotension from hyperglycaemic emergencies may mask the presence of hypertension. The mean age of our study population–may also partly explain the documented low prevalence of hypertension in this clinical scenario as ageing is associated with hypertension.

The duration of DM was associated with mortality outcomes as higher mortality rates were found in subjects with low duration of DM. Previously undetected cases of DM presenting in HEs may partly explain why this is so, since this group of patients fall under the low duration of DM category.

Electrolyte imbalances are the consequences of hyperglycemia, hyperosmolality, and acidosis. The biochemical parameters noted in this study were those of hypokalaemia. hyponatraemia, hypernatraemia and azotaemia and hyperkalaemia. These abnormalities occurred more in people with DKA except for hypernatraemia (HN) and hypokalaemia (HP) which occurred in higher percentages of subjects with HHS viz (12%,47% in HHS vs 2%,35% in DKA). Hypokalemia was the prevalent form of electrolyte imbalance observed in this Report. Hypokalaemia occurs as a a result of urinary losses in the face of a high osmotic gradient. Hyponatraemia, which was noted in about a third of subjects with types 1 and 2 DM often result from urinary losses and may be dilutional as water shifts extracellularly because of high serum osmolarity. Not surprising, serum hyperkalemia was seen only in a few patients and it's presence is explained by a shift of potassium from the intracellular to extracellular space because of acidosis from insulin deficiency and decreased renal tubular secretion. Azotemia which may be a resultant effect of volume contraction in patients with HEs was not objectively assessed as only urea levels of these patients were documented. However the presence of elevated urea levels was a prominent feature in our study subjects. Hypernatraemia, a rare feature of the HEs especially DKA may signify a response to reduction in circulating volume. The presence of hypotension, altered mentation and seizures in this report were infrequent occurring in 11 (10%), 26(23%) and 3(3%) of the subjects with HEs respectively. Seizures often occur in up to 25 percent of patients with hyperosmolar non ketotic state and may be generalized, focal, myoclonic jerking, or movement induced[16]. In this report, seizures not surprisingly occurred only in subjects with HHS. Hyperglycaemic emeregencies often are associated with very high mortality in sub-Saharan Africa, both in the treated patients and those who are presenting to hospital with diabetes for the first time[6]. The overall mortality rate of the HEs in this study was 20% and this figure is lower than those noted in some sub-Saharan African and Asian reports[7,12,15,17]. Mortality rates of HEs from sub-Saharan Africa and Asia range from 30–44% [7,11,14,16-19]. MacIssac et al [14] reported overall mortality rates of HEs as low as 4.8%. The estimated mortality rate for DKA range between 5–10% and that for HHS varies from 10 to 50%[10]. In this study, the case fatality of HHS at 35% was about twice that of DKA which was 18%. Our findings are in consonance with the general findings of higher case fatality rates of HHS compared with those of DKA [13,14]. Some of the predictive factors for fatal outcomes of the HEs in this report were sepsis, diabetes foot ulceration, low duration of DM and being elderly. The results from this report with regards to classification into types 1 and 2 should be interpreted with caution since objective classification of DM into these two types requires the assessment of C-Peptide levels which was not done.

### Limitations of the Study

1. We were unable to assess C peptide levels and this was as a result of financial constraints.

## Conclusion

Hyperglycaemic emergencies remain a major cause of concern in Nigerians with DM. Some of the determinants of fatal outcomes include DM foot ulcers, hypokalaemia and sepsis. These prognostic factors are readily modifiable and these should be focus points for health care providers.

## Competing interests

The authors declare that they have no competing interests.

## Authors' contributions

AO conceived of the study, participated in its design, acquisition of funding, drafting of manuscript and statistical analysis. JA contributed to study design and statistical analysis and acquisition of funding. CU contributed to drafting of manuscript and study design. OF participated in data collation and statistical analysis. All authors read and approved the final manuscript.

## Pre-publication history

The pre-publication history for this paper can be accessed here:


